# Prognostic significance of lymph node staging in esophageal squamous cell carcinoma after neoadjuvant chemoimmunotherapy

**DOI:** 10.3389/fonc.2026.1931817

**Published:** 2026-10-23

**Authors:** Herui Han, Xiaobin Hou, Yiming Liu, Wenhan Cai, Jiamei Jin, Xiangming Qiu, Zirui Zhu, Kai Zhao, Mingchuan Hu, Zhiqiang Xue

**Affiliations:** 1 Medical School of Chinese PLA, Beijing, China; 2 Department of Thoracic Surgery, The First Medical Centre, Chinese PLA General Hospital, Beijing, China; 3 School of Medicine, Nankai University, Tianjin, China

**Keywords:** clinical N stage, esophageal squamous cell carcinoma, neoadjuvant chemoimmunotherapy, pathological N stage, prognosis

## Abstract

**Background:**

Neoadjuvant chemoimmunotherapy (nCIT) has emerged as a standard treatment for locally advanced esophageal squamous cell carcinoma (ESCC). This study aimed to evaluate the independent prognostic value of postoperative pathological N stage (ypN) and to assess whether pre-treatment clinical N stage (cN) provides additional prognostic information in patients achieving ypN0.

**Methods:**

We retrospectively analyzed 194 ESCC patients who received nCIT followed by radical esophagectomy between January 2019 and December 2024. Patients were stratified into three groups: cN0/ypN0 (n=104), cN+/ypN0 (n=32), and ypN+ (n=58). Kaplan-Meier analysis and Cox proportional hazards models were used for survival analysis. Multivariable models were constructed using a clinically informed variable-selection strategy constrained by the number of events. For the three pairwise comparisons, unadjusted P values were compared against a Bonferroni-adjusted significance threshold of alpha=0.017 (0.05/3).

**Results:**

A total of 194 patients were enrolled, with 31 deaths and 37 recurrences during a median follow-up of 34.0 months (reverse Kaplan–Meier). ypN+ patients had significantly worse overall survival (OS) and recurrence-free survival (RFS) than ypN- patients (both Log-rank P<0.001). In the primary multivariable model, ypN+ was an independent adverse prognostic factor for OS (HR = 3.204, 95%CI: 1.563-6.568, P = 0.001) and RFS (HR = 3.780, 95%CI: 1.943-7.356, P<0.001), and remained robust across extended, cycle-adjusted, and landmark sensitivity analyses. Among the three groups, ypN+ showed significantly shorter OS compared with both ypN0 groups (P = 0.002 vs cN0/ypN0; P = 0.008 vs cN+/ypN0; both below the Bonferroni threshold) and shorter RFS compared with cN0/ypN0 (P<0.001). No significant difference was observed between cN0/ypN0 and cN+/ypN0 groups for OS (P = 0.259); the RFS comparison was nominally significant but did not survive Bonferroni correction (HR = 2.799, 95%CI: 1.048-7.479, unadjusted P = 0.040).

**Conclusion:**

Postoperative ypN stage is the core prognostic determinant in ESCC after nCIT. In patients achieving ypN0, pre-treatment cN did not demonstrate statistically significant additional prognostic discrimination in this cohort, although estimates were imprecise due to the small cN+/ypN0 subgroup and limited events. Postoperative risk assessment should be based primarily on ypN status; these findings require validation in larger, preferably multicenter, cohorts.

## 1 Introduction

Esophageal cancer is one of the most common gastrointestinal malignancies worldwide (1, 2). For locally advanced resectable esophageal squamous cell carcinoma (ESCC), neoadjuvant therapy followed by surgery has become the standard treatment approach. The CROSS trial (3, 4) and NEOCRTEC5010 trial (5) established the role of neoadjuvant chemoradiotherapy, while the JCOG1109 trial (6) demonstrated the efficacy of neoadjuvant chemotherapy. More recently, perioperative immunotherapy studies including Zheng et al. (7) and the NICE trial (8) have shown substantially improved pathological response rates and promising survival outcomes with neoadjuvant chemoimmunotherapy(nCIT), suggesting a potential shift toward the chemoimmunotherapy era. The therapeutic landscape continues to evolve rapidly, with ongoing investigation of induction chemoimmunotherapy combined with radiotherapy and other perioperative immunotherapy strategies (9).

Postoperative pathological N stage (ypN) is a core indicator for evaluating prognosis after neoadjuvant therapy. The American Joint Committee on Cancer (AJCC) 8th edition TNM staging system (10) introduced the ypTNM staging framework, and the prognostic value of nodal status after neoadjuvant therapy has been extensively validated in the chemotherapy and chemoradiotherapy eras (11, 12). However, with nCIT becoming an important treatment option for locally advanced ESCC, immune checkpoint inhibitors may alter lymph node metastasis regression patterns through systemic anti-tumor immune activation, potentially affecting the association between postoperative ypN and long-term prognosis. Therefore, re-validating the independent prognostic value of ypN in the nCIT context is essential for postoperative risk assessment in the chemoimmunotherapy era.

As nCIT converts a substantial proportion of clinically node-positive (cN+) patients to pathologically node-negative (ypN0), whether pre-treatment cN+ status retains residual prognostic disadvantage in patients achieving ypN0 remains unclear. Zanoni et al. (13) reported that cN+/ypN0 patients had significantly worse survival compared with cN0/ypN0 patients after neoadjuvant chemoradiotherapy (3-year survival: 59% vs. 84%). A recent meta-analysis by Su et al. (14) demonstrated no significant difference in OS between cN+/ypN0 and cN0/ypN0 groups in the subgroup of studies assessing pre-treatment nodal status by clinical imaging, suggesting limited supplementary prognostic value of pre-treatment cN status in patients achieving ypN0. However, these studies did not specifically focus on the nCIT setting.

Accordingly, this study was designed to address two key questions: first, to validate whether postoperative ypN status serves as an independent prognostic core indicator in ESCC patients after nCIT; and second, to assess whether pre-treatment clinical cN status based on imaging provides additional prognostic information on postoperative recurrence and survival risk in patients achieving ypN0.

## 2 Materials and methods

### 2.1 Study population

This single-center retrospective cohort study included ESCC patients who received nCIT followed by radical esophagectomy at the First Medical Center of Chinese PLA General Hospital between January 2019 and December 2024. Inclusion criteria were: (1) age 18–75 years; (2) clinically staged as cT1bN1-3M0 or cT2-4aN0-3M0 (AJCC 8th edition) based on contrast-enhanced chest CT, upper gastrointestinal contrast radiography, abdominal ultrasound or CT; (3) receipt of 2 or more cycles of taxane-based chemotherapy combined with a PD-1/PD-L1 inhibitor; (4) radical esophagectomy with systematic two-field or three-field lymph node dissection after nCIT; (5) postoperative pathological confirmation of ESCC. Exclusion criteria included: (1) concurrent other malignancies; (2) immunodeficiency, hematologic disorders, or autoimmune diseases; (3) distant metastasis during nCIT; (4) incomplete clinicopathological data.

### 2.2 Neoadjuvant therapy and surgery

All 194 patients received neoadjuvant chemoimmunotherapy, i.e., chemotherapy combined with immune checkpoint blockade. The chemotherapy backbone was taxane-based (paclitaxel or nab-paclitaxel) combined with a platinum agent (mainly cisplatin, carboplatin, or lobaplatin; nedaplatin or oxaliplatin in a small number of patients) or, in patients considered unsuitable for platinum, with S-1 (tegafur-gimeracil-oteracil). The immune checkpoint inhibitors used were mainly pembrolizumab, toripalimab, sintilimab, or adebrelimab, administered intravenously on day 1 of each 3-week cycle. Patients received 2–5 cycles of nCIT (median, 2 cycles). After completion of nCIT, patients were evaluated by a multidisciplinary team (MDT) and underwent radical esophagectomy with systematic lymph node dissection if deemed resectable. Postoperative (adjuvant) therapy was administered at the discretion of the treating team.

### 2.3 Grouping strategy

Patients were divided into three groups based on pre-treatment clinical cN status and postoperative pathological ypN status: (1) cN0/ypN0 group: clinically node-negative before nCIT and pathologically confirmed node-negative after surgery; (2) cN+/ypN0 group: clinically node-positive before nCIT but converted to pathologically node-negative after nCIT; (3) ypN+ group: pathologically confirmed persistent lymph node metastasis after surgery. Clinical staging was performed according to the AJCC 8th edition TNM criteria (10).

### 2.4 Assessment of pre-treatment clinical nodal status

Pre-treatment clinical nodal (cN) staging was performed before the initiation of nCIT using chest CT as the primary modality. On CT, a regional lymph node was considered suspicious for metastasis if its short-axis diameter was >=10 mm, or if, despite a short-axis diameter <10 mm, it exhibited morphological features suggestive of malignancy, including a rounded shape, loss of the fatty hilum, and heterogeneous or irregular enhancement with ill-defined borders. Endoscopic ultrasound (EUS) and whole-body PET-CT were performed in a subset of patients at the discretion of the treating team, rather than in all patients; when available, metabolic activity on PET-CT and EUS morphology were integrated into the final staging assessment. The final cN stage was determined independently by two physicians, and discordant interpretations were resolved by consensus. Because cN staging was completed before neoadjuvant treatment and surgery, the assessors were by design blinded to postoperative pathological findings.

### 2.5 Follow-up

Follow-up was conducted through outpatient visits, inpatient records, and telephone contact, with a data-censoring date of September 2025. Patients were evaluated every 3 months for the first year and every 6 months thereafter, including physical examination, contrast-enhanced CT of chest and upper abdomen, and tumor marker assessment. Overall survival (OS) was defined from the date of surgery to all-cause death or last follow-up. Recurrence-free survival (RFS) was defined from the date of surgery to disease recurrence, all-cause death, or last follow-up. Follow-up duration was the median follow-up estimated by the reverse Kaplan-Meier method.

### 2.6 Statistical analysis

Statistical analysis was performed using SAS 9.4 and R 4.4.4. Continuous variables were expressed as mean +/- standard deviation and categorical variables as frequency (percentage); group comparisons used one-way ANOVA and the chi-square or Fisher’s exact test, respectively. Survival curves were estimated by the Kaplan-Meier method and compared with the Log-rank test. Cox proportional hazards models were used to estimate hazard ratios (HR) with 95% confidence intervals (CI); the primary multivariable models included the clinically prespecified covariates ypN, cT, and ypT rather than variables selected by univariate screening, with model complexity constrained by event counts, and extended models additionally adjusting for age, sex, adjuvant therapy, and neoadjuvant cycle number were fitted as sensitivity analyses. For the three pairwise comparisons among groups, unadjusted P values were compared against a Bonferroni-adjusted threshold of alpha=0.017 (0.05/3). The proportional hazards assumption was examined using Schoenfeld residuals; when violated, piecewise hazard ratios and 36-month restricted mean survival time (RMST) were reported as complementary measures. To address potential immortal-time bias related to adjuvant therapy, a 3-month landmark analysis was performed excluding patients with events or censoring within 3 months after surgery. Two-sided P<0.05 was considered statistically significant unless otherwise specified.

## 3 Results

### 3.1 Clinicopathological characteristics

A total of 194 patients were enrolled, including 173 males (89.18%) and 21 females (10.82%), with a mean age of 60.6 +/- 6.9 years. Baseline characteristics differed significantly among the three groups in gender (P = 0.020), age (P = 0.002), cT stage (P = 0.003), cN stage (P<0.001), and ypT stage (P = 0.005), as did the number of neoadjuvant cycles (P = 0.042) and adjuvant therapy (P = 0.003) (**Table 1**); detailed treatment distributions are provided in **Supplementary Table S1**.

**Table 1:** Comparison of clinicopathological and treatment characteristics among the three groups (cN0/ypN0, cN+/ypN0, and ypN+).

Variable	Total (n=194)	cN0/ypN0 (n=104)	cN+/ypN0 (n=32)	ypN+ (n=58)	P
Gender					0.020
Female	21 (10.82)	14 (13.46)	6 (18.75)	1 (1.72)	
Male	173 (89.18)	90 (86.54)	26 (81.25)	57 (98.28)	
Age, years	60.6+/-6.9	62.1+/-6.7	57.6+/-7.5	59.5+/-6.4	0.002
cT stage					0.003
T1b-2	40 (20.62)	31 (29.81)	3 (9.38)	6 (10.34)	
T3-4	154 (79.38)	73 (70.19)	29 (90.63)	52 (89.66)	
cN stage					<0.001
cN-	110 (56.70)	104 (100.00)	0 (0.00)	6 (10.34)	
cN+	84 (43.30)	0 (0.00)	32 (100.00)	52 (89.66)	
ypT stage					0.005
ypT-	41 (21.13)	31 (29.81)	5 (15.63)	5 (8.62)	
ypT+	153 (78.87)	73 (70.19)	27 (84.38)	53 (91.38)	
ypN stage					<0.001
ypN-	136 (70.10)	104 (100.00)	32 (100.00)	0 (0.00)	
ypN+	58 (29.90)	0 (0.00)	0 (0.00)	58 (100.00)	
Neoadjuvant cycles					0.042
2 cycles	105 (54.12)	65 (62.50)	14 (43.75)	26 (44.83)	
>=3 cycles	89 (45.88)	39 (37.50)	18 (56.25)	32 (55.17)	
Adjuvant therapy					0.003
No	74 (38.14)	51 (49.04)	7 (21.88)	16 (27.59)	
Yes	120 (61.86)	53 (50.96)	25 (78.13)	42 (72.41)	

Data are n (%) or mean +/- standard deviation; P values from chi-square or Fisher’s exact tests across the three groups; cN, clinical N stage; ypN, postoperative pathological N stage; cT, clinical T stage; ypT, postoperative pathological T stage.

### 3.2 Independent prognostic value of ypN stage

The median follow-up was 34.0 months, estimated using the reverse Kaplan-Meier method. A total of 31 deaths and 37 recurrence events occurred: 12 deaths and 8 recurrences in the cN0/ypN0 group, 2 deaths and 8 recurrences in the cN+/ypN0 group, and 17 deaths and 21 recurrences in the ypN+ group.

The cN0/ypN0 and cN+/ypN0 groups were combined as the ypN- group (n=136) for comparison with the ypN+ group (n=58). Kaplan-Meier analysis showed a median OS of 49.0 months in the ypN+ group, which was not reached in the ypN- group. OS and RFS were significantly worse in the ypN+ group than in the ypN- group (both Log-rank P<0.001) (**Figure 1**).

**Figure 1:**
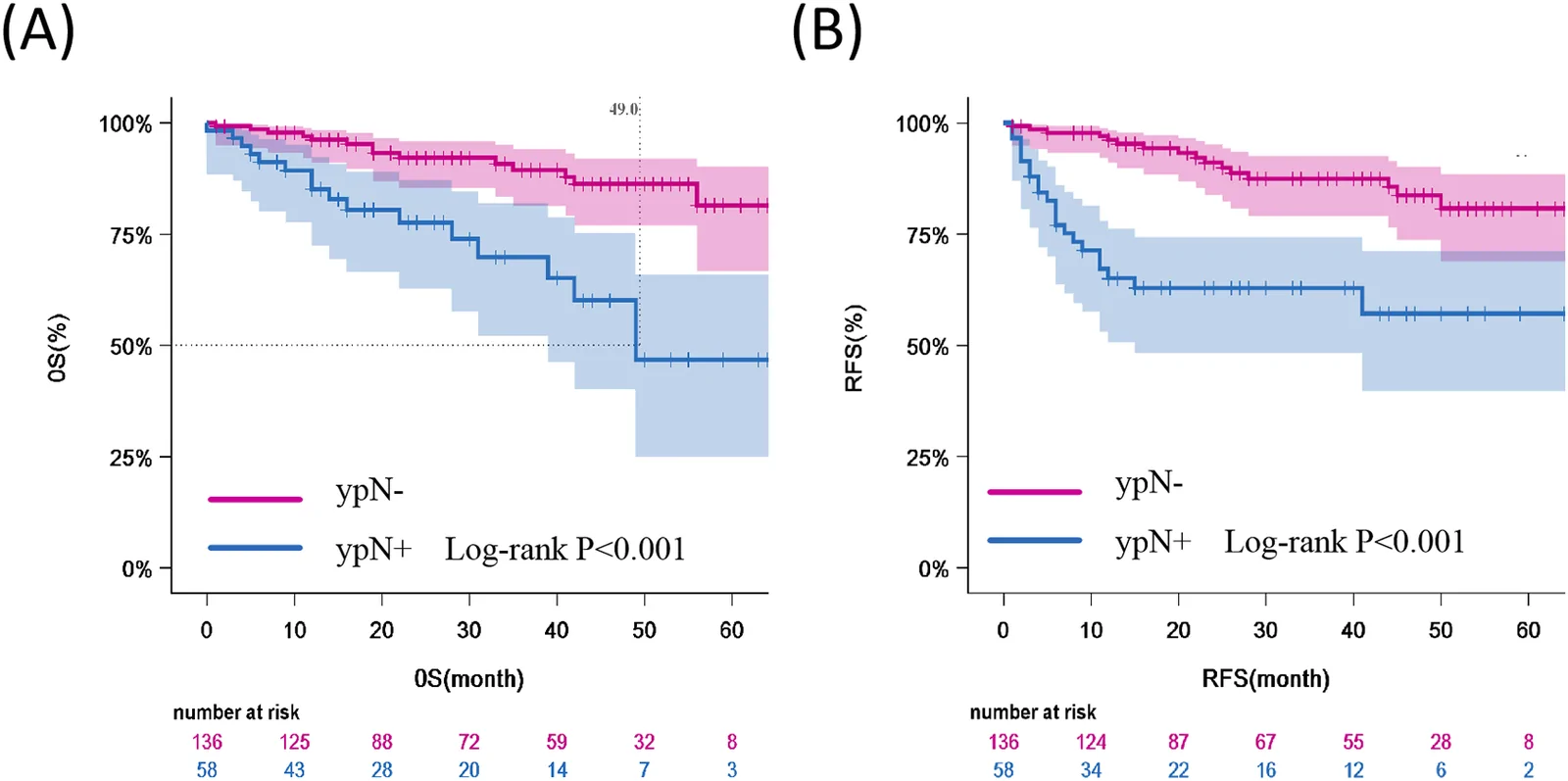
Kaplan-Meier curves for overall survival **(A)** and recurrence-free survival **(B)** according to postoperative nodal status (ypN- vs ypN+).

Univariate analysis showed that cT stage (T3-4) was associated with OS (HR = 4.688, 95%CI: 1.116-19.686, P = 0.034), ypT+ was associated with RFS (HR = 3.428, 95%CI: 1.052-11.171, P = 0.040), and ypN+ was associated with both OS (HR = 3.832, 95%CI: 1.882-7.803, P<0.001) and RFS (HR = 4.311, 95%CI: 2.239-8.300, P<0.001). In the primary multivariable model (ypN, cT, and ypT), ypN+ remained an independent adverse prognostic factor for OS (HR = 3.204, 95%CI: 1.563-6.568, P = 0.001) and RFS (HR = 3.780, 95%CI: 1.943-7.356, P<0.001), whereas cT and ypT were not independently significant (**Table 2**). The ypN+ association remained robust in extended models additionally adjusting for age, sex, adjuvant therapy, and neoadjuvant cycles (**Supplementary Table S2**).

**Table 2:** Univariate and multivariate Cox regression analyses of overall survival (OS) and recurrence-free survival (RFS).

Variable	OS Univariate HR (95% CI)	P	OS Multivariate HR (95% CI)	P	RFS Univariate HR (95% CI)	P	RFS Multivariate HR (95% CI)	P
Gender								
Female	1.000 (ref)				1.000 (ref)			
Male	2.039 (0.486-8.551)	0.330			1.563 (0.480-5.090)	0.459		
Age, years	1.002 (0.953-1.053)	0.941			0.956 (0.911-1.003)	0.067		
cT stage								
T1b-2	1.000 (ref)		1.000 (ref)		1.000 (ref)		1.000 (ref)	
T3-4	4.688 (1.116-19.686)	0.034	3.024 (0.691-13.232)	0.142	2.514 (0.889-7.114)	0.082	1.421 (0.481-4.203)	0.525
ypT stage								
ypT-	1.000 (ref)		1.000 (ref)		1.000 (ref)		1.000 (ref)	
ypT+	2.847 (0.865-9.373)	0.085	1.757 (0.517-5.968)	0.366	3.428 (1.052-11.171)	0.040	2.385 (0.701-8.114)	0.164
ypN stage								
ypN-	1.000 (ref)		1.000 (ref)		1.000 (ref)		1.000 (ref)	
ypN+	3.832 (1.882-7.803)	<0.001	3.204 (1.563-6.568)	0.001	4.311 (2.239-8.300)	<0.001	3.780 (1.943-7.356)	<0.001
Adjuvant therapy								
No	1.000 (ref)				1.000 (ref)			
Yes	0.643 (0.314-1.316)	0.227			1.274 (0.629-2.582)	0.501		

HR, hazard ratio; CI, confidence interval; ref, reference. Multivariable models included the prespecified covariates ypN, cT, and ypT.

The proportional hazards assumption was satisfied for all covariates in the primary OS model but was violated for ypN in the RFS model (Schoenfeld P = 0.010; **Supplementary Figures S1**, **S2**), indicating a time-varying effect: the excess recurrence risk of ypN+ patients was concentrated in the first postoperative year (0–12 months: HR = 11.05, 95%CI: 4.12-29.64, P<0.001; thereafter: HR = 0.76, 95%CI: 0.17-3.44, P = 0.723). Consistently, the 36-month restricted mean survival time was shorter in ypN+ than in ypN- patients (OS: 29.7 vs 34.2 months; RFS: 24.9 vs 33.7 months; **Supplementary Figure S3**).

### 3.3 Supplementary prognostic value of cN stage in ypN0 patients

Both OS and RFS differed significantly among the three groups (both Log-rank P<0.001; **Figure 2**). Pairwise comparisons (unadjusted P values compared against the Bonferroni threshold alpha=0.017) are summarized in **Table 3**: ypN+ patients had significantly shorter OS than both the cN0/ypN0 (HR = 3.237, 95%CI: 1.541-6.800, P = 0.002) and cN+/ypN0 (HR = 7.454, 95%CI: 1.708-32.520, P = 0.008) groups and significantly shorter RFS than the cN0/ypN0 group (HR = 6.353, 95%CI: 2.800-14.414, P<0.001). Between the two ypN0 groups, no significant difference was observed for OS (HR = 0.420, 95%CI: 0.093-1.897, P = 0.259), and the RFS comparison was nominally significant but did not survive correction (HR = 2.799, 95%CI: 1.048-7.479, unadjusted P = 0.040). The 3-year OS rates were 88.3% (95%CI: 78.1-93.9), 93.2% (75.4-98.3), and 69.8% (52.1-82.0), and the 3-year RFS rates were 93.1% (85.0-96.9), 73.4% (51.7-86.6), and 62.8% (48.3-74.3) in the cN0/ypN0, cN+/ypN0, and ypN+ groups, respectively (2-year rates and full estimates in **Supplementary Table S3**).

**Figure 2:**
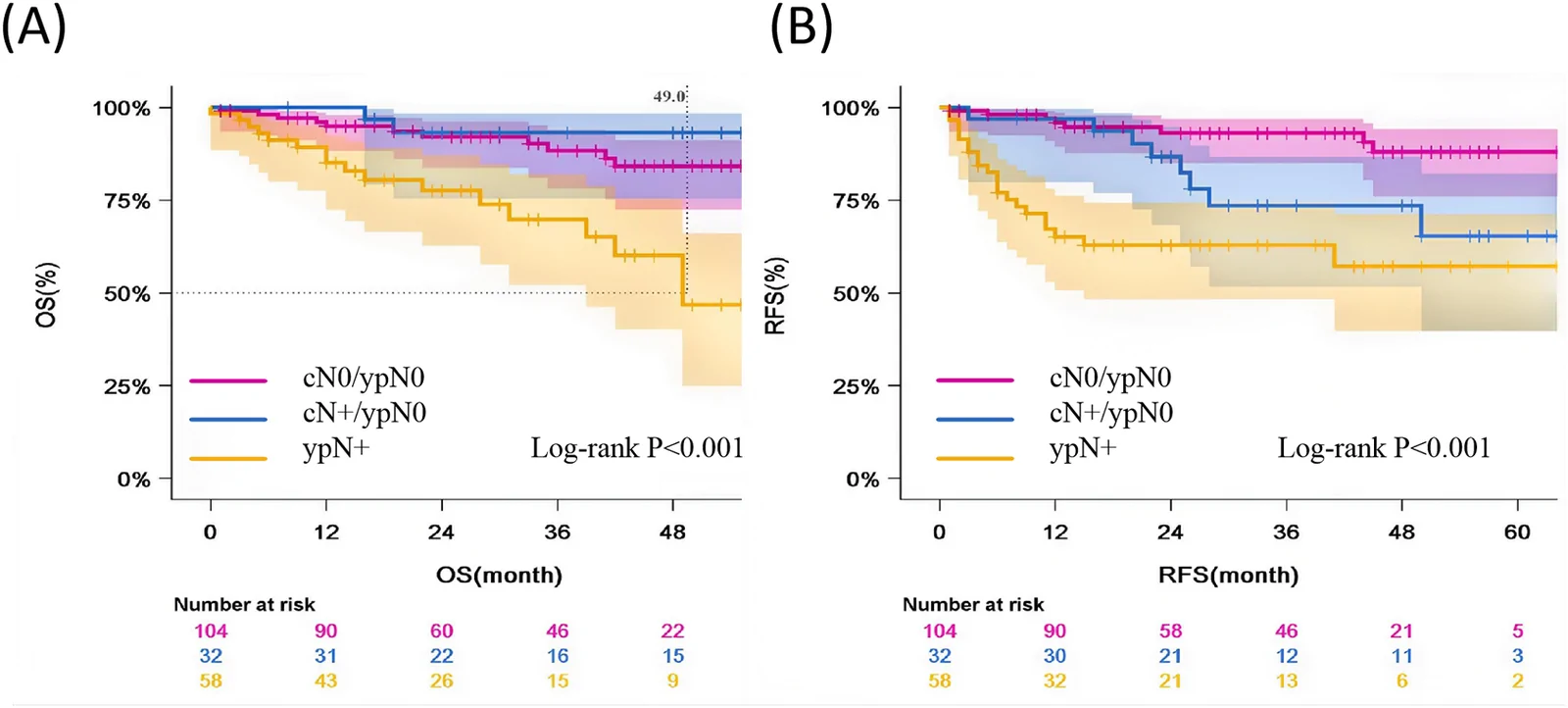
Kaplan-Meier curves for overall survival **(A)** and recurrence-free survival **(B)** among the cN0/ypN0, cN+/ypN0, and ypN+ groups.

**Table 3:** Pairwise comparisons of overall survival (OS) and recurrence-free survival (RFS) among the three groups.

Comparison	OS HR (95% CI)	P	RFS HR (95% CI)	P
cN0/ypN0 vs cN+/ypN0	0.420 (0.093-1.897)	0.259	2.799 (1.048-7.479)	0.040
cN0/ypN0 vs ypN+	3.237 (1.541-6.800)	0.002	6.353 (2.800-14.414)	<0.001
cN+/ypN0 vs ypN+	7.454 (1.708-32.520)	0.008	2.117 (0.932-4.810)	0.073

HR, hazard ratio; CI, confidence interval. Unadjusted P values were compared against a Bonferroni-adjusted threshold of alpha=0.017 (0.05/3).

In an exploratory multivariable analysis restricted to ypN0 patients (n=136; 14 OS and 16 RFS events) adjusting for adjuvant therapy and neoadjuvant cycles, cN+ status was not significantly associated with OS (HR = 0.556, 95%CI: 0.117-2.648, P = 0.461) but was associated with RFS (HR = 3.781, 95%CI: 1.312-10.890, P = 0.013) (**Supplementary Table S4**; **Supplementary Figures S4**,**S5**). Given the small number of events and wide confidence intervals, this adjusted RFS association should be interpreted as exploratory rather than definitive.

Taken together, pre-treatment cN status did not demonstrate statistically significant additional prognostic discrimination within the ypN0 population in this cohort; however, the small cN+/ypN0 subgroup (n=32) and limited events render these estimates imprecise, and absence of statistical significance should not be interpreted as equivalence.

### 3.4 Sensitivity analysis

To address potential immortal-time bias related to adjuvant therapy, a 3-month landmark analysis was performed excluding patients with events or censoring within 3 months postoperatively (n=187 for OS; n=180 for RFS). ypN+ remained independently associated with OS (HR = 3.833, 95%CI: 1.815-8.094, P<0.001) and RFS (HR = 4.550, 95%CI: 2.303-8.989, P<0.001), whereas adjuvant therapy was not (OS: HR = 0.737, P = 0.444; RFS: HR = 1.222, P = 0.607), indicating that adjuvant therapy was not a significant confounder of the ypN-survival association (**Supplementary Table S5**; **Supplementary Figures S6**, **S7**).

## 4 Discussion

This study addressed two questions in an ESCC cohort treated with nCIT. First, ypN+ was confirmed as an independent adverse prognostic factor for both OS and RFS (primary model: HR = 3.204 and 3.780, respectively), with consistent estimates across extended, cycle-adjusted, and landmark sensitivity analyses, establishing that ypN stage retains its core prognostic value in the chemoimmunotherapy era. Second, in patients achieving ypN0, pre-treatment clinical cN stage did not demonstrate statistically significant additional prognostic discrimination: neither the OS nor the RFS comparison between cN0/ypN0 and cN+/ypN0 met the Bonferroni-corrected threshold. However, the cN+/ypN0 subgroup comprised only 32 patients with 2 deaths and 8 recurrences, yielding imprecise effect estimates (e.g., RFS HR = 2.799, 95%CI: 1.048-7.479). Absence of statistical significance in this context reflects limited precision rather than equivalence, and our findings should be regarded as hypothesis-generating.

With nCIT becoming an important treatment option for locally advanced ESCC, immune checkpoint inhibitors may alter lymph node regression patterns through systemic anti-tumor immunity. CheckMate-577 (15) established adjuvant nivolumab after neoadjuvant chemoradiotherapy, early-phase nCIT studies such as NIC-ESCC2019 (16) demonstrated promising activity of neoadjuvant camrelizumab plus chemotherapy, and current guidelines (17) recommend neoadjuvant therapy for locally advanced ESCC, with perioperative immunotherapy continuing to evolve (9, 18, 19). Our multivariable analysis shows that ypN+ remains an independent adverse prognostic factor even in the nCIT context, consistent with evidence from the chemotherapy and chemoradiotherapy eras (3, 5, 20, 21) and with the long-established prognostic value of posttherapy pathologic stage (22). In line with this, Tang et al. (23) found comparable survival between natural N0 and downstaged N0 patients after neoadjuvant chemoradiotherapy (3-year OS: 76.7% vs. 79.5%, P = 0.794), and long-term CROSS results (24) confirmed the durability of chemoradiotherapy benefit; contemporary management is summarized in the ESMO guidelines (25). Throughout this study, cN and ypN are interpreted as prognostic factors; distinguishing prognostic from predictive biomarkers requires a comparator treatment arm or a treatment-by-biomarker interaction test, which our single-arm treated cohort cannot provide (26).

The prognostic significance of cN+/ypN0 status after neoadjuvant therapy remains debated. Zanoni et al. (13) reported worse survival in cN+/ypN0 than in cN0/ypN0 patients after neoadjuvant chemoradiotherapy (3-year survival: 59% vs. 84%), whereas Tang et al. (23) found comparable survival (3-year OS: 76.7% vs. 79.5%, P = 0.794), and a meta-analysis by Su et al. (14) found no significant OS difference between the two ypN0 subgroups when nodal status was assessed by clinical imaging. Our results were directionally consistent with this literature: no significant OS difference (P = 0.259) and a nominally significant RFS difference (P = 0.040) that did not survive Bonferroni correction, alongside an exploratory adjusted RFS association for cN+ (HR = 3.781, P = 0.013) based on only 16 events. We therefore cannot exclude a residual prognostic disadvantage carried by pre-treatment cN+ status, particularly for recurrence; adequately powered, preferably multicenter, validation cohorts or formal equivalence designs are needed.

Another finding of the study was the time-dependent nature of the ypN+ effect on recurrence. The proportional hazards assumption was violated for ypN in the RFS model (Schoenfeld P = 0.010), and piecewise analysis indicated that the excess recurrence risk of ypN+ patients was concentrated in the first postoperative year (0–12 months: HR = 11.05; >12 months: HR = 0.76). This temporal pattern is biologically plausible-residual nodal disease after nCIT may manifest as early recurrence-and carries a practical implication: if confirmed, ypN+ patients may benefit from intensified surveillance during the first postoperative year, whereas patients who remain recurrence-free beyond that window appear to have a subsequent risk comparable to ypN- patients. This observation should nonetheless be treated as a secondary, exploratory analysis.

Circulating tumor DNA (ctDNA)-based molecular residual disease detection offers a complementary avenue for postoperative risk stratification: postoperative ctDNA status has been shown to identify recurrence risk beyond pathological response and to inform adjuvant therapy decisions in esophageal cancer after neoadjuvant therapy (27, 28). Integrated models combining ypN with ypT or tumor regression grade, ctDNA status, and other clinical factors may outperform staging alone, and prospective studies incorporating serial ctDNA monitoring alongside ypTNM staging are warranted in the nCIT setting.

These findings should be translated cautiously into clinical practice. Our results support postoperative risk assessment based primarily on ypN status and identify ypN+ patients as a high-risk population that may warrant more intensive early surveillance, particularly during the first postoperative year. However, given the retrospective single-center design, the small cN+/ypN0 subgroup, and the limited follow-up, our data do not justify de-escalating surveillance in cN+/ypN0 patients; whether postoperative follow-up strategies can safely disregard pre-treatment cN status requires prospective multicenter validation.

Several limitations should be acknowledged. First, the single-center retrospective design carries inherent selection bias, and multicenter prospective validation is warranted. Second, the sample size is limited (n=194), particularly the cN+/ypN0 group (n=32; 2 deaths, 8 recurrences), which lacks power to detect modest differences; non-significant comparisons should not be interpreted as equivalence. Third, the median follow-up (34.0 months, reverse Kaplan-Meier method) is relatively short for OS assessment and few patients remained at risk toward the end of follow-up; OS findings should therefore be interpreted cautiously.Fourth, cN staging relied primarily on contrast-enhanced CT, with EUS and PET-CT in only a subset of patients; imaging-based nodal staging is subject to misclassification, which would attenuate between-group differences. Fifth, considerable treatment heterogeneity existed (ten immune checkpoint inhibitors, five platinum agents, and a platinum-free paclitaxel/S-1 regimen in 19.1% of patients), and the platinum distribution differed across groups (P<0.001); the sample size did not permit regimen-stratified analyses, although cycle adjustment did not materially change the results. Sixth, the initiation time of adjuvant therapy was not routinely recorded, precluding a time-dependent Cox model; the 3-month landmark analysis cannot fully exclude residual immortal-time bias. Finally, treatment-response depth indicators such as TRG or ctDNA were not incorporated and could be integrated with ypN in future studies.

## 5 Conclusions

Postoperative ypN stage is the core prognostic determinant in ESCC patients after nCIT, with ypN+ conferring an approximately three- to four-fold increase in the hazards of death and recurrence, concentrated in the first postoperative year for recurrence. In patients achieving ypN0, pre-treatment clinical cN stage did not demonstrate statistically significant additional prognostic discrimination in this cohort; however, estimates were imprecise due to the small cN+/ypN0 subgroup and limited events, and these hypothesis-generating findings require validation in larger multicenter prospective cohorts before informing surveillance de-escalation.

## Data Availability

The data analyzed in this study is subject to the following licenses/restrictions: The datasets are not publicly available due to patient privacy and ethical restrictions. Data are available from the author upon reasonable request. Requests to access these datasets should be directed to Herui Han, 15922527654@163.com.
