# Supplementary material for: Prognostic significance of lymph node staging in esophageal squamous cell carcinoma after neoadjuvant chemoimmunotherapy
Source: Front Oncol. 2026 Oct 23;16:1931817. doi: 10.3389/fonc.2026.1931817 (PMC13739558; doi:10.3389/fonc.2026.1931817)
Supplement: Supplementary file 1 [file Table1.docx]

**Supplementary Material**

**Prognostic significance of lymph node staging in esophageal squamous cell carcinoma after neoadjuvant chemoimmunotherapy**

Herui Han, Xiaobin Hou, Yiming Liu, Wenhan Cai, Jiamei Jin, Xiangming Qiu, Zirui Zhu, Kai Zhao, Mingchuan Hu, Zhiqiang Xue

*Corresponding author: Zhiqiang Xue. E-mail address: xuezhiqiang301@126.com*

# Supplementary Tables

**Table S1. Detailed distributions of neoadjuvant regimens, immune checkpoint inhibitors, and adjuvant therapy among the three groups (cN0/ypN0, cN+/ypN0, and ypN+).**

| Variable | Total (n=194) | cN0/ypN0 (n=104) | cN+/ypN0 (n=32) | ypN+ (n=58) | P |
| --- | --- | --- | --- | --- | --- |
| Platinum agent |  |  |  |  | <0.001 |
| Cisplatin | 63 (32.47) | 42 (40.38) | 4 (12.50) | 17 (29.31) |  |
| Carboplatin | 44 (22.68) | 30 (28.85) | 1 (3.13) | 13 (22.41) |  |
| Lobaplatin | 47 (24.23) | 19 (18.27) | 11 (34.38) | 17 (29.31) |  |
| Nedaplatin | 2 (1.03) | 0 (0.00) | 0 (0.00) | 2 (3.45) |  |
| Oxaliplatin | 1 (0.52) | 0 (0.00) | 0 (0.00) | 1 (1.72) |  |
| None (paclitaxel + S-1) | 37 (19.07) | 13 (12.50) | 16 (50.00) | 8 (13.79) |  |
| Immune checkpoint inhibitor |  |  |  |  | 0.239 |
| Pembrolizumab | 69 (35.57) | 31 (29.81) | 14 (43.75) | 24 (41.38) |  |
| Toripalimab | 51 (26.29) | 25 (24.04) | 14 (43.75) | 12 (20.69) |  |
| Sintilimab | 28 (14.43) | 18 (17.31) | 2 (6.25) | 8 (13.79) |  |
| Adebrelimab | 23 (11.86) | 15 (14.42) | 0 (0.00) | 8 (13.79) |  |
| Tislelizumab | 9 (4.64) | 6 (5.77) | 1 (3.13) | 2 (3.45) |  |
| Camrelizumab | 6 (3.09) | 4 (3.85) | 0 (0.00) | 2 (3.45) |  |
| Nivolumab | 3 (1.55) | 2 (1.92) | 0 (0.00) | 1 (1.72) |  |
| Durvalumab | 2 (1.03) | 2 (1.92) | 0 (0.00) | 0 (0.00) |  |
| Sugemalimab | 2 (1.03) | 1 (0.96) | 1 (3.13) | 0 (0.00) |  |
| Serplulimab | 1 (0.52) | 0 (0.00) | 0 (0.00) | 1 (1.72) |  |
| Adjuvant therapy regimen |  |  |  |  |  |
| No adjuvant therapy | 74 (38.14) | 51 (49.04) | 7 (21.88) | 16 (27.59) |  |
| ICI-containing regimen | 86 (44.33) | 38 (36.54) | 19 (59.38) | 29 (50.00) |  |
| Chemotherapy alone | 6 (3.09) | 1 (0.96) | 0 (0.00) | 5 (8.62) |  |
| Regimen not documented | 28 (14.43) | 14 (13.46) | 6 (18.75) | 8 (13.79) |  |

*Note. Data are presented as n (%). P values refer to the distribution across the three groups (chi-square or Fisher’s exact test). All patients received neoadjuvant chemoimmunotherapy; the “None (paclitaxel + S-1)” category denotes a platinum-free chemotherapy backbone.*

**Table S2. Extended multivariable Cox models for overall survival (OS) and recurrence-free survival (RFS).**

| Variable | Model 1 OS HR (95% CI) | P | Model 1 RFS HR (95% CI) | P | Model 2 OS HR (95% CI) | P | Model 2 RFS HR (95% CI) | P |
| --- | --- | --- | --- | --- | --- | --- | --- | --- |
| ypN+ (vs ypN-) | 3.360 (1.588, 7.112) | 0.001 | 3.720 (1.868, 7.410) | <0.001 | 3.505 (1.640, 7.490) | 0.001 | 3.881 (1.930, 7.803) | <0.001 |
| cT3-4 (vs cT1b-2) | 3.258 (0.738, 14.395) | 0.119 | 1.400 (0.476, 4.115) | 0.541 | 3.459 (0.776, 15.428) | 0.104 | 1.501 (0.507, 4.444) | 0.463 |
| ypT+ (vs ypT-) | 1.654 (0.478, 5.728) | 0.427 | 2.354 (0.694, 7.982) | 0.169 | 1.547 (0.439, 5.448) | 0.497 | 2.197 (0.641, 7.534) | 0.211 |
| Age, per year | 1.021 (0.969, 1.076) | 0.429 | 0.967 (0.919, 1.017) | 0.193 | 1.020 (0.968, 1.075) | 0.457 | 0.968 (0.920, 1.018) | 0.203 |
| Male (vs female) | 1.348 (0.300, 6.052) | 0.696 | 0.830 (0.242, 2.852) | 0.768 | 1.327 (0.295, 5.966) | 0.712 | 0.826 (0.240, 2.844) | 0.762 |
| Adjuvant therapy (yes vs no) | 0.553 (0.266, 1.146) | 0.111 | 1.116 (0.549, 2.267) | 0.762 | 0.539 (0.259, 1.121) | 0.098 | 1.087 (0.533, 2.215) | 0.818 |
| Neoadjuvant cycles >=3 (vs 2) | - | - | - | - | 0.726 (0.332, 1.590) | 0.424 | 0.699 (0.350, 1.398) | 0.312 |

*Note. HR, hazard ratio; CI, confidence interval. Model 1 included ypN, cT, ypT, age, sex, and adjuvant therapy; Model 2 additionally included the number of neoadjuvant cycles (2 vs >=3).*

**Table S3. Two- and three-year survival rates by group (Kaplan-Meier estimates with 95% confidence intervals).**

| Group | 2-year OS (95% CI) | 3-year OS (95% CI) | 2-year RFS (95% CI) | 3-year RFS (95% CI) |
| --- | --- | --- | --- | --- |
| cN0/ypN0 (n=104) | 92.0% (83.7-96.2) | 88.3% (78.1-93.9) | 93.1% (85.0-96.9) | 93.1% (85.0-96.9) |
| cN+/ypN0 (n=32) | 93.2% (75.4-98.3) | 93.2% (75.4-98.3) | 86.7% (68.3-94.8) | 73.4% (51.7-86.6) |
| ypN+ (n=58) | 77.6% (62.7-87.1) | 69.8% (52.1-82.0) | 62.8% (48.3-74.3) | 62.8% (48.3-74.3) |

*Note. OS, overall survival; RFS, recurrence-free survival; CI, confidence interval.*

**Table S4. Adjusted multivariable Cox models restricted to ypN0 patients (n=136; 14 OS events and 16 RFS events).**

| Variable | OS HR (95% CI) | P | RFS HR (95% CI) | P |
| --- | --- | --- | --- | --- |
| cN+ (vs cN0) | 0.556 (0.117, 2.648) | 0.461 | 3.781 (1.312, 10.890) | 0.013 |
| Adjuvant therapy (yes vs no) | 0.523 (0.178, 1.538) | 0.239 | 0.757 (0.243, 2.357) | 0.631 |
| Neoadjuvant cycles >=3 (vs 2) | 0.422 (0.090, 1.983) | 0.275 | 0.235 (0.051, 1.086) | 0.064 |

*Note. HR, hazard ratio; CI, confidence interval. Schoenfeld residual tests.*

**Table S5. Sensitivity analysis: 3-month landmark Cox regression analysis excluding patients with events or censoring within 3 months postoperatively.**

| Variable | OS HR (95% CI) | P | RFS HR (95% CI) | P |
| --- | --- | --- | --- | --- |
| ypN stage |  |  |  |  |
| ypN- | 1.000 (ref) |  | 1.000 (ref) |  |
| ypN+ | 3.833 (1.815, 8.094) | <0.001 | 4.550 (2.303, 8.989) | <0.001 |
| Adjuvant therapy |  |  |  |  |
| No | 1.000 (ref) |  | 1.000 (ref) |  |
| Yes | 0.737 (0.338, 1.608) | 0.444 | 1.222 (0.569, 2.626) | 0.607 |

*Note. HR, hazard ratio; CI, confidence interval; ref, reference.*

# Supplementary Figures

**Figure S1. Schoenfeld residual diagnostics for the primary multivariable overall survival (OS) model (covariates: ypN, cT, ypT).** Scaled Schoenfeld residuals are plotted against time for (A) cT stage, (B) ypT stage, and (C) ypN stage. The proportional hazards (PH) assumption was satisfied for all covariates (cT: P=0.936; ypT: P=0.688; ypN: P=0.455).

Figure S1

**Figure S2. Schoenfeld residual diagnostics for the primary multivariable recurrence-free survival (RFS) model (covariates: ypN, cT, ypT).** Scaled Schoenfeld residuals are plotted against time for (A) cT stage, (B) ypT stage, and (C) ypN stage. The PH assumption was satisfied for cT (P=0.348) and ypT (P=0.139) but was violated for ypN (P=0.010), indicating a time-varying effect of ypN on recurrence; piecewise hazard ratios and 36-month restricted mean survival time are therefore reported in the main text as complementary effect measures.

Figure S2

**Figure S3. Restricted mean survival time (RMST) at 36 months by ypN status.** (A) Overall survival (OS): RMST was 34.2 months (95%CI: 33.0-35.3) in the ypN- group versus 29.7 months (95%CI: 26.7-32.8) in the ypN+ group. (B) Recurrence-free survival (RFS): RMST was 33.7 months (95%CI: 32.4-34.9) in the ypN- group versus 24.9 months (95%CI: 21.0-28.8) in the ypN+ group. RMST is presented as a complementary, assumption-free effect measure in view of the non-proportional hazards of ypN for RFS.

Figure S3

**Figure S4. Schoenfeld residual diagnostics for the adjusted overall survival (OS) model restricted to ypN0 patients (covariates: cN, adjuvant therapy, and number of neoadjuvant cycles).** Scaled Schoenfeld residuals are plotted against time for (A) adjuvant therapy, (B) cN status, and (C) neoadjuvant cycles.

Figure S4

**Figure S5. Schoenfeld residual diagnostics for the adjusted recurrence-free survival (RFS) model restricted to ypN0 patients (covariates: cN, adjuvant therapy, and number of neoadjuvant cycles).** Scaled Schoenfeld residuals are plotted against time for (A) adjuvant therapy, (B) cN status, and (C) neoadjuvant cycles.

Figure S5

**Figure S6. Schoenfeld residual diagnostics for the 3-month landmark overall survival (OS) model (covariates: ypN and adjuvant therapy).** Scaled Schoenfeld residuals are plotted against time for (A) ypN stage and (B) adjuvant therapy. The PH assumption was satisfied for ypN (P=0.770) but showed evidence of violation for adjuvant therapy (P=0.018); the corresponding hazard ratios should therefore be interpreted as averaged effects over the follow-up period.

Figure S6

**Figure S7. Schoenfeld residual diagnostics for the 3-month landmark recurrence-free survival (RFS) model (covariates: ypN and adjuvant therapy).** Scaled Schoenfeld residuals are plotted against time for (A) ypN stage and (B) adjuvant therapy. The PH assumption was violated for ypN (P=0.005), consistent with the time-varying recurrence effect observed in the primary RFS model, and was satisfied for adjuvant therapy (P=0.957).

Figure S7
